# Should Israel be concerned by the high proportion of medical care paid for privately: comments from a U.S. perspective

**DOI:** 10.1186/s13584-016-0068-5

**Published:** 2016-03-15

**Authors:** Stuart Altman

**Affiliations:** The Heller School for Social Policy and Management, Brandeis University, 415 South St, Waltham, MA 02453 USA

## Abstract

As a frequent visitor to Israel, I am keenly aware of the concerns of many Israelis that limits in government funding is forcing a high proportion of the country’s medical care to be paid by private sources. Frequently used statistics suggested that close to 40 % of health care is paid for by private funds and that this generates inequalities in terms of access to needed services. The results of a recent IJHPR paper by Engelcin-Nissan and Shmueli cast some doubts on these concerns although they do suggest some degree of inequality in access to needed care. The authors suggest that a better measure of the proportion of private uses of care is not 39 % but about 15 %. And that for some essential medical services it is much lower; in particular, it is less than 10 % for primary care. On the other hand, the study indicates that, in 2009, 15 % of hospitalizations had some private funding. Moreover, a related study has indicated that in 2014 60 % of surgeries were supported by private funds. The authors raise additional concerns that sicker individuals and those with higher income are more likely to use private financing.

Whether this level of private spending and its concentration on sicker and higher income individuals violates the commitment of equity and fairness is up to the citizens of Israel. For those of us in the U.S. we only wish our level of inequality was so low. In making the decision on what Israel should do about its inequality it would be helpful to understand why individuals use private funding for services that are covered by the national health insurance system. And, most importantly does using a different source of funds (private versus public) impact on the health outcomes of the care involved. This issue is particularly relevant with respect to the very high use of private financing for surgeries.

## Background

For someone like myself who has spent much of my professional life trying to reduce the amount of inequality in the U.S. healthcare financing system [1], I am sympathetic to those who are concerned about inequality in access to care. With that said, we must also guard against being too rigid in requiring that everyone have equal access to all services or that one type of financing (private versus public) is necessarily better. In countries like Israel where substantial differences in income exist there inevitably will be differences in access to care and the use of more private spending by higher income individuals. The question is how much inequity in use or financing should be allowed, for what types of services, and the consequences of these inequalities. The results of the recent IJHPR article by Engelchin-Nissan and Shumeli [2] can be a useful first step in answering these questions.

There has been a great deal of discussion and concern in Israel about the fact that almost 40 % of all health expenditures in the country are not financed by government (i.e. paid by some form of private insurance or out-of-pocket payments excluding co-payments) and that this is one of the highest private spending rates of any OECD country. The implication of this high private spending amount is that it generates inequalities in access to care and poorer medical outcomes by those with limited incomes. The authors of this study believe, however, that this 40 % rate is misleadingly high. They suggest that a more meaningful measure in relation to these equity concerns is the proportion of expenditures of services included in the national package of benefits that have some private financing. Thus, services such as dental, mental health and nursing home care were excluded from their analysis. Given limitations with data availability the authors were required to focus on a “private services use rate” as defined as those covered services “used” by individuals that were not totally paid for by national health insurance sources (through the sick funds) as a proportion of all covered services “used”. These include services that were financed in part through national health insurance and in part by private sources (but beyond the nominal co-payment levels for pharmaceuticals and visits to specialists).

For some purposes, a use proportion may be preferable to one which focuses on expenditures but I would be careful in comparing the 40 % private expenditure rate to the 15 % private use rate found by Engelchin-Nissan and Shmueli. As we know different medical procedures have very different costs and it is highly unlikely that the use rate would be similar to the expenditure rate. Nevertheless, I do believe the information generated in this study is a useful addition to the national debate on whether more private financing (or less government financing) generates inequalities in terms of uses of medical care and poorer outcomes for those that rely entirely on government funds. It would also be of interest to see how Israel would compare with other countries in terms of the “use” measure employed by Engelchin-Nissan and Shmueli.

What I found most positive about the results of the study is that it appears that the government financed Israeli health system does a good job in supporting basic health services like primary care. Under their definition, in 2009 private payments were involved in less than 10 % of primary care services used and exceed a 30 % use rate only for what many might consider less essential paramedical care. For example, some form of private financing is used to pay for 69 % of occupational services used and 51 % of speech therapy. It should be understood that these paramedical care services could be of value for patients who need them but not at the same level as those services covered by the national insurance system.

What is more troublesome about their findings is the 15 % private use rate for hospitalizations and the possibility that, as suggested by a related study [3], in 2014 23 % of hospitalizations and 60 % of surgeries used private funding, and that such private funding is more likely to be used by higher income and better educated patients. The authors too are concerned about these findings. They state “This (finding) obviously violates the principle that equally sick persons should enjoy equal care”. (I must admit I have a problem with this definition of equity. Whether we should be concerned with what source of funds is used to pay for care should be related primarily to the type of care provided by the different sources and the differential in medical outcomes. It is possible that if all patients had to use public funding for all medical care it could lead to poorer quality care for all income groups. In other words, equity for what purpose?)

Of further concern to the authors is that those with chronic medical conditions are significantly more likely to use privately financed care. A question in my mind is whether this occurs because the publicly financed system is too restrictive or the population involved feels the need to seek additional or different services even when these services may not add substantially to the ultimate health outcomes. Again what is not measured in this study is whether those that use only public funded care receive less care or that the care they receive is poorer quality or leads to inferior health outcomes.

As an outsider, I do not think it is my purview to question how another society should pay for an important service such as healthcare. There are many good reasons for a healthcare system to rely on government generated funds. I also believe there may be good reasons for at least a portion of a country’s healthcare services to be financed privately (given the need for other government supported services or to keep taxes lower) [1].

Implicit in the paper is that the use of privately generated funds in Israel has grown too large and its use violates the principle of equity. This is a fair concern. Particularly if the growth in private funding is the result of government unduly restricting what it provides to support its health system. But just because some individuals use private financing doesn’t mean they are necessarily getting better care. They may be using private funds for more convenient care or for other personal reasons. Even if they are receiving more care, it is also unclear whether it really helps them generate better health.

## Conclusions

The importance of the findings of this paper is to understand how the citizens of Israel are using their privately supported healthcare services. Here I think those who support the national health system should take some solace. For what most consider fundamental healthcare services the publically supported system seem to be doing an okay job. True non-government health spending in Israel is quite significant (39 %) but as the paper argues a more meaningful measure is that only 15 % of direct medical care used that is included in the national basket of covered services is paid for privately (excluding dental, mental health and long-term care). What is troubling, however, is that even though the highest proportion of privately funded care used is for non-basic health services such as occupational health, private funds do support a substantial amount of hospital care, particularly surgery.
